# Molecular allergy diagnosis using pollen marker allergens and pollen panallergens: Five patterns seen in multiple test reactions to pollen extracts 

**DOI:** 10.5414/ALX02238E

**Published:** 2021-05-27

**Authors:** Jörg Kleine-Tebbe, Juliane Ackermann-Simon, Gerald Hanf

**Affiliations:** Allergy and Asthma Center Westend, Outpatient Clinic & Research Center Hanf, Ackermann and Kleine-Tebbe, Berlin, Germany

**Keywords:** skin prick test (SPT), IgE, allergen-specific IgE test, pollen, major allergen, pan-allergen, cross reactivity, primary sensitisation, profilin, polcalcin, cyclophilin

## Abstract

Polysensitizations to tree, grass, and weed pollen are found in ~ 20% of pollen-allergic individuals. They are often based on broad IgE cross-reactivities to pollen panallergens belonging to highly conserved protein families: 1. profilins, 2. polcalcins (calcium-binding proteins in pollen), 3. cyclophilins. They represent highly conserved cross-reactive minor allergens present in all pollen species, but also in plant foods and other organisms. Despite being rarely clinically relevant they can hamper allergy diagnostic tests with extracts. In this situation, molecular allergy diagnosis is able to distinguish broad cross-reactivity due to allergen-specific IgE to pollen panallergens (i.e. profilins Bet v 2 or Phl p 12; polcalcins Bet v 4 or Phl p 7; and, in the future, cyclophilins Bet v 7 or Ole e 15) from primary IgE sensitizations to so-called marker allergens represented by important pollen major allergens: Bet v 1 for the birch and beech family (*Fagales*), Ole e 1 for olive and ash (*Oleaceae*), Phl p 1 for temperate climate grasses (*Poaceae*), Art v 1 for mugwort (*Artemisia*), Amb a 1 for Ambrosia species (*Ambrosia*). Five typical cases (A – E) with positive skin prick test results to tree, grass, and weed pollen extracts demonstrate typical patterns of IgE sensitization with a variable impact of pollen panallergens: A – profilins, B – polcalcins, C – profilins and polcalcins, D – presumably cyclophilins, E – primary polysensitization to tree, grass, and weed pollen without interference from profilins or polcalcins. Differences between pollen extract-based skin prick test diagnosis and molecular allergen-specific IgE testing are explained using the presented concept. This approach allows to reduce the number of allergen extracts – presuming they are also clinically relevant – for allergen immunotherapy (i.e., only tree and/or grass pollen extracts), particularly in pollen-polysensitized patients.

## Introduction 

Allergy diagnosis is difficult in polysensitized pollen-allergic patients. Test reactions in all pollen groups (tree, grass, and weed pollen) are often based on broad cross-reactions. The reason for this are IgE sensitizations against pollen panallergens [1, 2], which are probably present in all pollen regardless of their allergological significance. The so-far known representatives, all minor allergens with great similarity within their families, are evolutionarily strongly conserved proteins:

- Profilins, in all pollen and many plant-based foods (e.g., melon, banana, etc.) [3], but also in other organisms. In Central Europe, ~ 15 – 20% of all pollen-allergic patients are IgE positive [1], in Southern Europe, however, the proportion is significantly higher [3].
- Polcalcins, Ca^++^-binding proteins (CBPs) in all types of pollen [4], ~ 5% of pollen-allergic patients in Central Europe are IgE positive [1].
- Cyclophilins, in pollen [5] and plant-based foods [6], but also in other organisms [5, 7]. The frequency of IgE sensitization to cyclophilins among pollen-allergic patients is unclear.

Most broad cross-reactions between tree, grass, and weed pollen can probably be explained by panallergens (personal observations). Pollen extracts are not very helpful for diagnostic workup, neither in skin prick testing nor in specific IgE testing, since they lose their analytical specificity [8] as all plant extracts potentially contain the extremely cross-reactive panallergens. When determining IgE antibodies against natural plant allergens (e.g., pollen extracts or naturally purified pollen allergens such as nPhl p 4), it must also be taken into account that many proteins are glycoproteins and as such carry CCD epitopes, which cause clinically irrelevant, serological in-vitro cross-reactivity. Fortunately, skin prick tests with pollen extracts are not falsified by low-affinity anti-CCD IgE. 

In this situation, specific IgE tests against marker allergens are useful [8]. These are important major allergens, e.g., of a certain pollen-producing plant, representative of a homologous group [9]. If the selected marker allergens were produced recombinantly (e.g., rBet v 1) and do not have CCD epitopes, significantly increased specific IgE confirms a primary sensitization while negative IgE can safely rule it out. Often only a few important pollen groups or pollen-producing plants remain after molecular allergy diagnosis. The results make it easier to decide which pollen extracts are suitable for allergen immunotherapy, if there is a clinical indication. 

Five selected cases involving pollen panallergens show exemplary patterns and the results of molecular allergy diagnosis. 

## Materials and methods 

### Open history-taking questions 

Skin prick testing using commercial allergen extracts (ALK-Abelló, Hamburg, Germany; Allergopharma, Reinbek, Germany). 

Additional palm tree pollen: a) profilin fraction (naturally purified), b) residual fraction containing polcalcin (ALK-Abelló Laboratory, Madrid, Spain). 

Reading after 15 minutes and documentation of the mean wheal diameter. 

Serological IgE testing using the ImmunoCAP Singleplex system: Total IgE, allergen-specific IgE against pollen panallergens (one profilin and one polcalcin each) and pollen major allergens (Table 1, right column). 

## Patients 

Five selected patients (A – E) with seasonal symptoms plus

- positive skin prick test to representatives of all pollen groups (tree, grass, and weed pollen) plus
- results of targeted specific IgE tests against single pollen allergens (“components”).

**Clinical symptoms**

Patient A: For more than 20 years – mild seasonal allergic symptoms in early summer (May, June) with itchy eyes, sneezing, runny nose, and sometimes itchy palate. 

Patient B: For ~ 12 years and getting worse – itchy eyes, sneezing, runny nose, rarely bronchial symptoms. Season: February to March/April at the latest; less in June until July. Symptom improvement after subcutaneous allergen immunotherapy (tree and grass pollen mixture for 5 years). 

Patient C: For the past 15 years in spring – runny nose, itchy eyes, eye redness, hardly any sneezing, symptoms start exacerbating again in May. For the past 6 years – increasingly perennial cold-like symptoms with frequent sneezing, runny nose, and nasal congestion with known mite allergy. 

Patient D: For the last 8 years – increasingly itchy eyes, sneezing fits, runny nose in spring (season: February to March/April at the latest), sometimes difficulty breathing (without wheezing). Since approximately 2017 – allergy symptoms not only in spring, but, less pronounced, until August. Sores on the oral mucosa after eating peaches and many other (raw) fruits. 

Patient E: Since adolescence – seasonal allergic symptoms with itching, tearing, and redness of the eyes, sometimes swellings of the eyes, sneezing fits, runny nose, itchy palate and ears in spring and summer (length of the season unclear). 

## Results 

The sensitizations of the five selected patients with pollen allergy show heterogeneous results in the skin prick and allergen-specific IgE tests (Table 1). When interpreting the results, deviations between the skin prick tests using extracts and the IgE results against pollen panallergens and marker allergens were particularly taken into account. 

Patient A: Despite positive skin tests for various types of pollen, sensitization to *Fagales* pollen (neg. Bet v 1 sIgE), ash pollen (neg. Ole e 1 sIgE), and mugwort pollen (neg. Art v 1 sIgE) could be ruled out with great certainty. Positive sIgE (>10% of total IgE) against grass pollen major allergens (groups 1 and 5) shows a predominant sensitization to grass pollen. The slightly increased sIgE against Phl p 12 (timothy grass profilin) corresponds to the skin test result and is probably responsible for the positive prick tests for various pollen extracts. Conclusion: Predominant grass pollen sensitization with weak profilin sensitization and concomitant cross-reactions to various pollen extracts. 

Patient B: Some of the multiple pollen sensitizations seen in the skin prick test (e.g., ash, mugwort, and ragweed pollen) are not confirmed by specific IgE testing against the corresponding marker allergens (Ole e 1, Art v 1, Amb a 1), as they turned out negative. There are clear sensitizations against grass and tree pollen (*Fagales*). In this case, many positive skin prick tests are based on sIgE against polcalcin (pos. Phl p 7-sIgE). Conclusion: Tree pollen (*Fagales*) and grass pollen sensitization with multiple pollen cross-reactions through additional polcalcin sensitization. 

Patient C: The markedly increased total IgE as an indication of an increased disposition to develop atopic eczema also results in significantly increased specific IgE values against the individual allergens. Molecular diagnosis confirms predominant tree pollen (*Fagales*) and grass pollen sensitization, shows comparatively low Ole e 1 sIgE and no sensitizations to Amb a 1 and Art v 1. Sensitizations to two pollen panallergens (profilin and polcalcin) provide an explanation for the positive test results for *all* pollen extracts tested. Conclusion: Predominant tree pollen (*Fagales*) and grass pollen sensitization with multiple cross-reactions to pollen extracts due to combined profilin and polcalcin sensitization. 

Patient D: Despite many positive skin prick test reactions to pollen extracts, only a massive sensitization against tree pollen (*Fagales*) can be confirmed (> 28% Bet v 1 sIgE in relation to total IgE). Despite positive reactions to the polcalcin-containing extract, the IgE test does not confirm sensitization against polcalcin (or profilin). Conclusion: Predominant birch/*Fagales* pollen sensitization with numerous pollen cross-reactions and without involvement of the pollen panallergens profilin or polcalcin. 

Patient E: Numerous positive skin prick test reactions against tree, grass, and weed pollen were confirmed by targeted testing of the corresponding major allergens (positive sIgE against Bet v 1, Ole e 1, Phl p 1/Phl p 5, Amb a 1, and Art v 1), but without detection of sIgE against Phl p 7 or Phl p 12. Conclusion: Genuine pollen polysensitization against birch/*Fagales,* ash, grass, and weed pollen (ragweed and mugwort) without the involvement of the pollen panallergens profilin or polcalcin. 

## Discussion 

**Figure 1.**
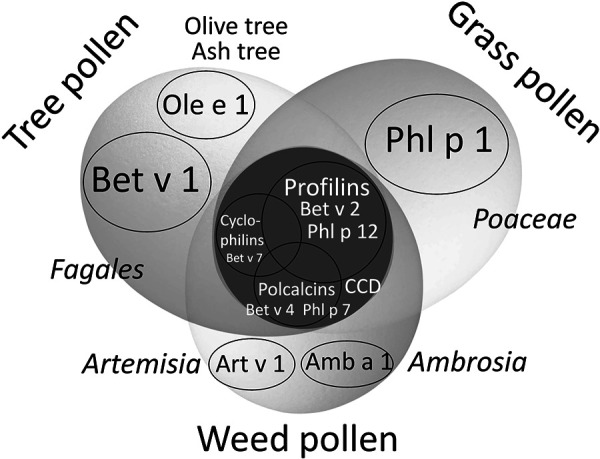
Updated propeller model for allergy diagnosis in case sensitization to pollen panallergens is suspected. Representative major allergens (“marker allergens” see propeller blades) for specific IgE diagnostics and highly cross-reactive panallergens (center of propeller) as a potential cause of multiple cross-reactions (positive tree, grass, and weed pollen) when using (pollen) extracts.

Molecular diagnostic workup with pollen allergens is actually only useful in *polysensitized* pollen-allergic patients. In this context, it is important to distinguish between primary sensitizations and broad cross-reactions caused by pollen panallergens (Figure 1). In the case of positive test reactions to only one group of pollen (trees *or* grasses *or* weeds (Figure 1, propeller blades) or two groups (trees *and* grasses, trees *and* weeds, grasses *and* weeds), an involvement of pollen panallergens is unlikely, since the latter are highly conserved proteins occurring in all of types of pollen (Figure 1, center of propeller). Nevertheless, there is usually some variation in test reactions to different pollen extracts, since the proportion of pollen panallergens is often unclear and variable. 

Profilins, and probably also cyclophilins, are also present in almost all (raw) vegetable foods; profilins are particularly found in, e.g., melons, but also in all other fruits and vegetables, tree nuts, and legumes. Another cyclophilin, Ara h 18, was recently detected in peanut. In patients with grass pollen allergy, it was responsible for serological cross-reactions that could not be explained with other peanut allergens identified so far (e.g., CCD or profilins) [6]. Due to their thermal and acid instability, profilins (and probably also cyclophilins) mostly only cause allergic symptoms when raw plant products are ingested, and symptoms are limited to the mouth and throat. 

In Central Europe, the development of sensitization to profilin and/or polcalcin is probably mostly based on a pronounced grass pollen sensitization. Theoretically, however, other regionally important pollen species from trees or weeds (e.g., birch in Scandinavia, ambrosia/ragweed in North America, mugwort in China) can also lead to an additional sensitization to pollen panallergens. 

The individual clinical relevance of these ubiquitous minor allergens can often not be clarified based on patient history. In a small case series, Spanish pollen-allergic patients with proven profilin sensitization showed predominantly positive reactions after nasal and/or bronchial provocation with 99% purified profilin (Pho d 2) extracts from date palm pollen [10]. The latter are currently not available for routine diagnostic workup outside of Spain. Due to the high development costs and regulatory hurdles, in-vivo extracts with pollen panallergens are unlikely to be introduced even in the future. As an alternative, a pollen variety that is considered non-allergenic in Central Europe (such as palm pollen) could serve as an indicator of the potential involvement of pollen panallergens in the prick test if the reaction is positive. 

The five presented cases represent five typical patterns (% of all patients with pollen allergy):

- Broad pollen cross-reactivity due to profilin sensitization (approx. 15%);
- Broad pollen cross-reactivity due to polcalcin sensitization (approx. 5%);
- Universal cross-reactivity against all types of pollen due to combined profilin and polcalcin sensitization (approx. 2%);
- Broad pollen cross-reactivity due to cyclophilin sensitization (frequency unknown);
- Genuine polysensitization against important pollen species without broad cross-reaction in the absence of sensitization against pollen panallergens (rare finding).

In all cases (A – E), molecular allergy diagnosis, in contrast to pollen extract-based diagnostic workup, allows to identify primary predominant sensitization against important pollen species. Moreover, primary sensitizations to mugwort and ragweed can be clearly separated using Art v 1 and Amb a 1. The clinical relevance of these sensitizations has to be clarified based on patient history or provocation testing. The extent to which allergen immunotherapy (AIT) is less successful when additional sensitization to pollen panallergens is present (A – D) has never been prospectively investigated, especially since their content in most AIT extracts is unknown. The arguments for the targeted use of specific major allergens in sIgE tests are more the demand for a precise allergen-specific diagnosis and avoidance of unsuitable pollen species when an AIT is planned. 

This is especially true for case D, in which the broad cross-reactivity against many pollen is obviously not based on profilin- or polcalcin-specific IgE. With regard to the positive skin prick test for the polcalcin-containing palm pollen fraction (Table 1, case D), it must be taken into account that other pollen panallergens are likely to be found in this extract. The most likely candidate is a cyclophilin, which is an extremely conserved ubiquitous protein and has been identified not only in plant cells but also in animal cells, molds, and even bacteria [11]. In case D, specific IgE against Bet v 7, a cyclophilin in birch pollen [5], could be responsible for the broad cross-reactivity between numerous pollen plants that are not closely botanically related. Thus, besides profilins and polcalcins, cyclophilins represent the third pollen panallergen family that is potentially responsible for ubiquitous cross-reactions between botanically unrelated pollen species [7]. Unfortunately, no recombinantly produced pollen cyclophilins, such as Bet v 7, Ole e 15, or Ara h 18, are currently available for molecular sIgE diagnostic workup. As with profilins and polcalcins, one representative would probably suffice due to the high intra-family cross-reactivity. 

In case E, the clinical relevance of the proven primary sensitizations to major allergens of important pollen species is unclear due to the incomplete patient history. Symptom calendar and provocation tests would be suitable means for the targeted selection of the relevant pollen species for a future AIT, if clinically indicated and desired. 

**Figure 2.**
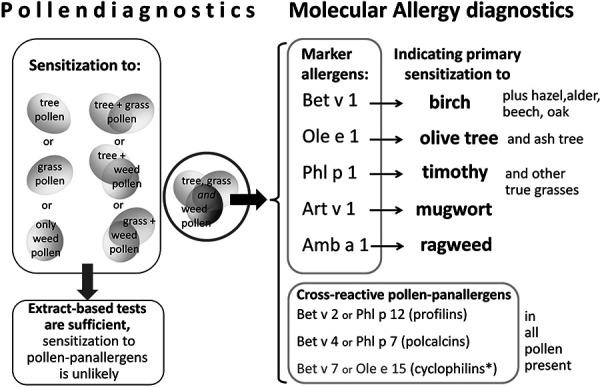
Diagnostic algorithm for polysensitized patients with pollen allergy. Sensitizations against one or two groups of pollen (top left box) do usually not involve pollen panallergens. Diagnostic workup (e.g., skin prick test, specific IgE test) with pollen extracts is then sufficient to determine and distinguish important sensitizations (bottom left box). Only sensitizations to all three pollen groups (circle) can be based on highly cross-reactive pollen panallergens. Specific IgE tests using marker allergens (top right box) and one representative of each of the potentially involved pollen panallergens (bottom right box) allow reliable differentiation: 1. Primary sensitization against important allergen sources, yes or no? 2. Detectable cross-reactions caused by pollen panallergens, which *family* and which *origin* (pollen species)? Sensitizations against pollen panallergens (basically pollen minor allergens) are mostly caused by pollen with high seasonal exposure and the resulting broad and predominant sensitizations (e.g., against grass pollen) as reflected by the high specific IgE against the associated marker allergen (e.g., Phl p 1). *So far, no pollen cyclophilin is available for sIgE diagnostic workup so that not all cases of pollen polysensitization can be determined (see patient D). For allergen immunotherapy, if indicated, only pollen extracts with proven primary sensitization (positive marker allergen) and clinical relevance would be considered in polysensitized patients.

All presented cases show sensitizations against pollen extracts from trees, grasses, and weeds. With this pattern, molecular allergy diagnosis allows to distinguish between primary sensitizations and broad cross-reactions caused by pollen panallergens (profilins, polcalcins, and, in the future also, cyclophilins). The diagnostic algorithm used (Figure 2) thus allows more precise allergen-based diagnoses, since confusing polysensitizations can be resolved in most cases with standard diagnostic workup for pollen. Subsequently, even supposed “multi-allergic” patients can often be treated with one or a few allergen extracts/preparations as part of an AIT. To what extent its success is diminished by additional sensitizations to pollen panallergens is not yet clear due to the lack of prospectively collected data. 

## Conclusion 

Complex sensitization patterns (against tree, grass, and weed pollen in ~ 1/5 of all patients with pollen allergy) can be successfully distinguished using molecular allergy diagnosis. Broad cross-reactions are frequently caused by pollen panallergens (profilins, polcalcins, and possibly cyclophilins). The detection of primary sensitizations against important marker allergens facilitates the selection of the clinically relevant extracts for AIT in these patients. 

## Funding 

None. 

## Conflict of interest 

J. Kleine-Tebbe reports personal fees from AllergenOnline (Nebraska, USA), Allergy Therapeutics, Allergopharma, ALK-Abelló, AstraZeneca, Bencard, HAL Allergy, LETI, Sanofi, Springer International Publishers, ThermoFisher Scientific, and Thieme Publishers, Germany; has received grants and personal fees from GSK, Lofarma, Novartis, Regeneron, and Stallergenes-Greer; and has received personal fees and non-financial support from the German Society of Allergy and Clinical Immunology; all outside the submitted work. 

**Table 1.** Skin prick test and specific IgE results of 5 patients with pollen allergy (patients A – E).

	**Patient A**		**Patient B**		**Patient C**		**Patient D**		**Patient E**		
Skin prick test	Wheal ø	Total IgE	Wheal ø	Total IgE	Wheal ø	Total IgE	Wheal ø	Total IgE	Wheal ø	Total IgE	IgE tests
Controls	(mm)	(kU/L)	(mm)	(kU/L)	(mm)	(kU/L)	(mm)	(kU/L)	(mm)	(kU/L)	Total IgE
Histamin (10 mg/mL)	4	70	5	680	6	2110	6	129	6	251	
NaCl solution (0.9%)	0		0		0		1		0		
	Wheal ø	specific IgE	Wheal ø	specific IgE	Wheal ø	specific IgE	Wheal ø	specific IgE	Wheal ø	specific IgE	
Pollen extracts:	(mm)	(kU_A_/L)	(mm)	(kU_A_/L)	(mm)	(kU_A_/L)	(mm)	(kU_A_/L)	(mm)	(kU_A_/L)	Allergen molecules:
Birch	4	< 0.1	7	11	7	127	9	37	10	12.5	Bet v 1 (major all.)
Hazel	4		7		10		10		12		
Alder	4		5		8		7		8		
Oak	2		4		7		7		10		
Ash	4	< 0.1	5	< 0.1	7	11	5	< 0.1	7	4	Ole e 1 (major all.)
Grasses	6	5.7	7	45	5	> 100	7	< 0.1	8	5	Phl p 1 (major all.)
		2.2				88		< 0.1		4	Phl p 5 (major all.)
Palm/polcalcin and others	0	< 0.1	7	20	10	15	4	< 0.1	0	< 0.1	Phl p 7 (polcalcin)
Palm/Profilin nPho d2	6	0.33	0	< 0.1	10	11	0	< 0.1	0	< 0.1	Phl p 12 (profilin)
Ragweed	0	< 0.1	4	< 0.1	3	< 0.1	0	< 0.1	7	4	Amb a 1
Mugwort	3	< 0.1	5	< 0.1	6	< 0.1	0	< 0.1	8	8.5	Art v 1
Nettle	0		8		8		0		2		
Goosefoot	0		6		10		5		2		
Ribwort plantain	6		8		5		4		5		

Leftmost column: pollen-based diagnosis, corresponding left columns (gray): prick test results; rightmost column: molecular allergy diagnosis, corresponding right columns (white): IgE results.
